# One Size Does Not Fit All: Face Emotion Processing Impairments in Semantic Dementia, Behavioural-Variant Frontotemporal Dementia and Alzheimer’s Disease Are Mediated by Distinct Cognitive Deficits

**DOI:** 10.3233/BEN-2012-0349

**Published:** 2011-12-29

**Authors:** Laurie A. Miller, Sharpley Hsieh, Suncica Lah, Sharon Savage, John R. Hodges, Olivier Piguet

**Affiliations:** ^1^Neuropsychology UnitRoyal Prince Alfred HospitalCamperdownNSWAustralia; ^2^School of PsychologyUniversity of SydneySydneyNSWAustralia; ^3^Neuroscience Research AustraliaSydneyNSWAustralia; ^4^School of Medical SciencesUniversity of New South WalesSydneyNSWAustralia

**Keywords:** Semantic dementia, behavioural-variant frontotemporal dementia, naming, identity matching

## Abstract

Patients with frontotemporal dementia (both behavioural variant [bvFTD] and semantic dementia [SD]) as well as those with Alzheimer's disease (AD) show deficits on tests of face emotion processing, yet the mechanisms underlying these deficits have rarely been explored. We compared groups of patients with bvFTD (*n* = 17), SD (*n* = 12) or AD (*n* = 20) to an age- and education-matched group of healthy control subjects (*n* = 36) on three face emotion processing tasks (Ekman 60, Emotion Matching and Emotion Selection) and found that all three patient groups were similarly impaired. Analyses of covariance employed to partial out the influences of language and perceptual impairments, which frequently co-occur in these patients, provided evidence of different underlying cognitive mechanisms. These analyses revealed that language impairments explained the original poor scores obtained by the SD patients on the Ekman 60 and Emotion Selection tasks, which involve verbal labels. Perceptual deficits contributed to Emotion Matching performance in the bvFTD and AD patients. Importantly, all groups remained impaired on one task or more following these analyses, denoting a primary emotion processing disturbance in these dementia syndromes. These findings highlight the multifactorial nature of emotion processing deficits in patients with dementia.

## 1. Introduction

Lesion and functional neuroimaging studies have demonstrated specific neural substrates that underlie the recognition of emotions [1–5]. Extra-striate networks involving the amygdala [6,7], insula [2,8,9] and ventral striatum [1,10] have been identified. Not surprisingly, patients with frontotemporal dementia (FTD), which involves atrophic changes in many of these same brain regions, show impairments on tests of face emotion processing [11–17]. Such deficits have been documented in the two main subtypes of FTD: behavioural-variant (bvFTD) and semantic dementia (SD) [18,19]. The prototypical presentation in patients

ISSN 0953-4180/12/$27.50 © 2012 – IOS Press and the authors. All rights reserved with bvFTD and SD involves changes in social interactions, with bvFTD associated with disinhibited and inappropriate behaviour and SD associated with a decrease in empathy (e.g., [20–23]). Deficits in face emotion processing have been postulated to contribute to the socially inappropriate behaviour demonstrated by patients with FTD (e.g., [14,15,24]).

Patients with Alzheimer's disease (AD), however, show similar deficits on tests of face emotion processing to those seen in FTD [25,26], yet AD patients typically do not show the same behaviour changes in the early stages of their disorders. Hence, it has been proposed that patients with different types of dementia may fail emotion processing tasks for different reasons. For example, FTD patients have been reported to show more variability in face emotion processing when tested on multiple occasions compared to AD patients [13] and the authors postulated that an underlying problem with impulse control might contribute to their deficient scores. Furthermore, impairments in visual perception, such as those experienced by AD patients [27], and/or language deficits, such as those seen in the FTD subtypes [28], are likely to contribute to the documented emotion processing deficits. This view is further supported by the fact that AD, bvFTD and SD patients show different regional patterns of brain atrophy and cortical thinning [19,29,30].

Depending on the task demands, measures of emotion processing invariably involve additional skills such as face perception, expressive speech and/or semantic knowledge pertaining to an emotion label. This study aimed to investigate face emotion judgment integrity and identify the contribution of other cognitive deficits present in AD, bvFTD and SD to face emotion processing. This aim was achieved by using control tasks matched to the face emotion tasks in terms of presentation and response mode (but differing in stimuli) to partial out the possible effects of perceptual, semantic and response mode deficits. We hypothesised that patients with AD, bvFTD or SD would all exhibit emotion processing deficits; however, in each group, this disturbance would be related to different underlying cognitive deficits.

## 2. Methods

### *2.1. Participants*

Forty-nine patients meeting clinical diagnostic criteria for FTD or AD [22,31] were recruited from the Frontotemporal Dementia Research Group in Sydney. All patients were seen by the same experienced behavioural neurologist (J.R.H.) and diagnosis was based on clinical assessment, comprehensive neuropsychological assessment and presence of brain atrophy on structural MR. Patients presenting with behavioural features of bvFTD in the absence of brain atrophy or progression, also known as phenocopy bvFTD [32], were excluded. Three SD patients with predominant right-sided temporal lobe atrophy were also excluded, as this small number of cases prevented separate analyses for this group. Other exclusion criteria included: Presence of other neurological disease; diagnosis of major depression, schizophrenia, obsessive compulsive disorder or substance abuse; and score on the Mini-mental state examination (MMSE) [33] $< 19/30$. Thirty-six age- and education-matched healthy control (HC) participants were recruited from the patients' families and friends, or from the local area. Participants were excluded if any of the following criteria were present: significant history of psychiatric or neurological condition, history of substance abuse or medications with CNS side effects, and a MMSE $< 27/30$.

### *2.2. Materials*

We used three tests of emotion processing and three control measures.

#### *2.2.1. Emotion processing tasks*

(1) Ekman 60 (Emotion labeling task) – This task assesses recognition and labeling of six basic emotions (Anger, Disgust, Fear, Happiness, Sadness, Surprise) using 60 stimuli from the Pictures of Facial Affect series [34]. Each image was presented on a computer screen for 5 s in a pseudorandom order and participants were instructed to select the label that best described the emotional expression. The emotion labels were present throughout testing and selection was untimed. Participants responded either by using the mouse to click the appropriate label, pointing to the label, or saying their response (depending of their preference), for the researcher to record. No feedback on response accuracy was provided.
(2) Emotion Matching Task – This task assesses the ability to match facial emotions across different faces and requires no overt verbal labelling. Images from the NimStim stimulus set were used (www.macbrain.org). Faces were cropped so that nonfacial features (e.g., hair) were removed using Adobe Photoshop Version CS2 to reduce cues for discrimination. Participants viewed 42 pairs of faces in greyscale on a white background on a computer screen, one pair at a time. For each pair, participants were instructed to determine whether the two faces displayed the same emotion. The two faces presented were always from two different individuals. The task was untimed.

(3) Emotion Selection Task – This task measures the ability to choose a facial emotion based on a verbal label. In this task, participants viewed arrays of seven faces of the same person displaying the six basic emotions, as well as a neutral expression and were instructed to point to the face corresponding to the label spoken by the examiner (e.g., "Point to the HAPPY face"). Faces and positions of the emotions within the array varied across trials. Forty-two items were presented and faces were selected from the NimStim stimulus set. A blank screen separated each trial and the task was untimed.

*2.2.2. Control tasks*

(1) BNT15 – This picture naming task contains 15 line drawings from the Boston Naming Test [35]. The number of items named spontaneously (prior to phonemic cueing) was recorded.
(2) Face Matching Task – This task is similar to the Emotion Matching Task described above and also contains 42 pairs of faces from the NimStim face set. Each pair was presented one at a time and participants were instructed to determine whether the two faces belonged to the same individual. The task was untimed and each pair of faces always showed different emotions.
(3) Word-Picture Matching Task – This is a 30-item semantic word-picture matching task [36] modelled on the Cambridge semantic battery [37]. On each trial, participants viewed an array of seven coloured photographs comprising one target and six related foils, and were instructed to point to the item that corresponds to a verbal label (e.g., "Point to the RHINOCEROS"). The task was untimed.

### 2.3. Procedure

Patients were tested on the face processing and control tasks described above as part of a large clinical and experimental neuropsychological assessment during their initial work-up that also included the MMSE and Addenbrooke's Cognitive Examination-Revised (ACE-R; [38]). These scores were used to examine group-matching for more general cognitive abilities. The tests that are part of this study were given in a random order.

The Southeastern Sydney and Illawarra Area Health Service and the University of New South Wales ethics committees approved the study. Participants, or their person responsible, provided informed consent. All participants volunteered their time but were reimbursed for travel costs.

### 2.4. Statistical analyses

Group differences were investigated using one-way analysis of variance (ANOVA) tests followed by Tukey post hoc tests where appropriate, or Chi-squared tests for dichotomous variables. Relations between face emotion processing and control task performance were examined using Pearson correlation test. When correlations were significant, Analyses of Covariance (ANCOVA) were carried out to determine whether group differences on the face emotion processing tasks remained after using the control tasks as covariates. All results significant at the $p < 0.05$ level are reported here.

## 3. Results

The groups were matched for sex, age and education, and patient groups were also matched for disease duration and dementia severity (all $p$s $> 0.15$) as measured by the Clinical Dementia Rating (CDR) [39] (Table 1). Significant group differences were present on the cognitive screening measures (MMSE: $F = 32.3$, $p < 0.001$, and ACE-R: $F = 83.4$, $p < 0.001$), with all three patient groups being impaired relative to the control group (all $p$s $< 0.05$). On both measures, the SD and AD groups obtained lower scores than the bvFTD group (both $p$s $< 0.01$). Not surprisingly, the SD group also performed worse than the AD group ($p < 0.001$) on the ACE-R given its larger language component.

Significant differences among groups were present on all Face Emotion Processing tasks (Ekman 60: $F = 14.5$, $p < 0.001$; Emotion Matching: $F = 11.8$, $p < 0.001$; Emotion Selection: $F = 16.0$, $p < 0.001$) (Fig. 1). On each task, the patient groups performed significantly below controls (all $p$s $< 0.001$), but performance across patient groups did not differ (all $p$s $> 0.05$).

Table 1
Demographic characteristics for the healthy control and patient groups

| | HC ($n$ = 36) | bvFTD ($n$ = 17) | SD ($n$ = 12) | AD ($n$ = 20) |
|---|---|---|---|---|
| Sex (F/M) | 12/24 | 1/16 | 4/8 | 6/14 |
| Age (yrs) | 65 ± 5.8 | 61 ± 7.8 | 62 ± 8.2 | 65 ± 8.0 |
| Education (yrs) | 14 ± 2.8 | 12 ± 3.4 | 12 ± 3.1 | 12 ± 3.0 |
| Disease duration (months) | N/A | 44 ± 28.4 | 49 ± 12.0 | 36 ± 14.9 |
| MMSE (max: 30) | 29.0 ± 0.9 | 27.7 ± 1.3 | 24.7 ± 2.6 | 25.3 ± 2.7 |
| ACE-R (max: 100) | 95 ± 3.0 | 86 ± 4.0 | 62 ± 10.4 | 77 ± 10.1 |
| CDR (max: 3) | N/A | 0.74 ± 0.31 | 0.59 ± 0.30 | 0.89 ± 0.37 |

*Note.* Values are mean ± standard deviation. HC = healthy controls; bvFTD = behavioural-variant frontotemporal dementia; SD = semantic dementia; AD = Alzheimer's disease.; N/A = not applicable.

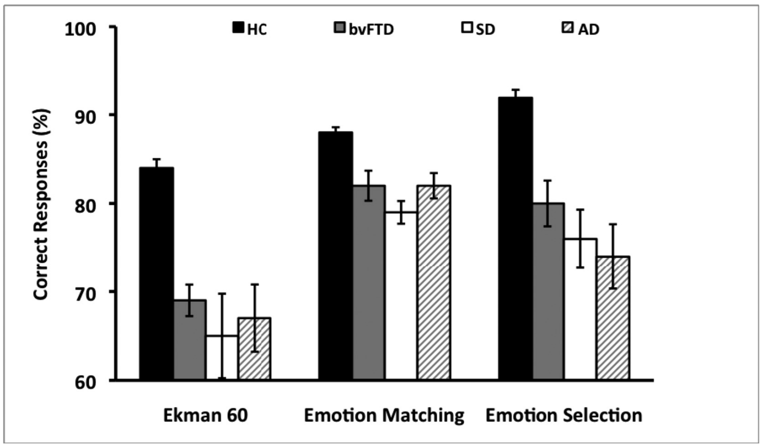

Fig. 1. Results of ANOVAs for Face Emotion Processing Tasks. All three patient groups were impaired relative to healthy controls (HC), but no group differences were present between patients with behavioural-variant frontotemporal dementia (bvFTD), semantic dementia (SD) or Alzheimer's disease (AD).

In addition, ANOVAs indicated that groups also differed significantly on the control tasks (BNT15: $F = 152$, $p < 0.001$; Face Matching: $F = 7.4$, $p < 0.001$; Word-Picture Matching: $F = 29.8$, $p < 0.001$) (Table 2). On the BNT15, post hoc analyses indicated that the SD and AD named significantly fewer items than the bvFTD group and the HC group (all $p$s $< 0.05$). Moreover, the SD group was impaired compared to the AD group ($p$s $< 0.001$). On Face Matching, the bvFTD and AD groups correctly matched fewer faces than controls ($p < 0.01$), but the three patient groups did not differ from each other. Post hoc analyses of the Word-Picture Matching task indicated that both the SD and AD patients were impaired relative to the HC group and the SD group was also impaired relative to the bvFTD and AD groups ($p$s $< 0.001$).

As predicted, performance on each of the Emotion Processing Tasks was significantly correlated with its respective control task (Ekman 60 and BNT15: $r = 0.40$, $p < 0.001$; Emotion Matching and Face Matching: $r = 0.50$. $p < 0.001$; Emotion Selection and Word-Picture Matching: $r = .47$, $p < 0.001$).

Results of the ANCOVAs are presented in Fig. 2. Group differences remained on all three face processing measures (Ekman 60: $F = 10.6$, $p < 0.001$; Emotion Matching: $F = 12.5$; $p < 0.001$; Emotion Selection: $F = 13.7$, $p < 0.001$). Post hoc tests showed that both the bvFTD and AD groups remained impaired on the Ekman 60 and the Emotion Selection tasks compared to the control group. On Emotion Matching, only the SD group remained impaired relative to controls.

## 4. Discussion

Disturbance of emotion processing is a core feature of the two most common subtypes of FTD (bvFTD and SD) and is also common in AD, although such deficits may be due to different underlying cognitive impairments. In this study, we found that while all three

Table 2
Performance (percent correct) on the control tasks for healthy control and patient groups

| | HC | bvFTD | SD | AD |
|---|---|---|---|---|
| BNT15 (max = 15) | 98 ± 4.0 | 90 ± 9.1 | 22 ± 17.5 | 79 ± 14.2 |
| Face Matching (max = 42) | 88 ± 9.2 | 73 ± 15.7 | 82 ± 13.8 | 76 ± 13.5 |
| Word-Picture Matching (max = 30) | 98 ± 3.1 | 94 ± 7.2 | 66 ± 21.9 | 86 ± 10.7 |

*Note:* Values are mean ± standard deviation. HC = healthy controls; bvFTD = behavioural-variant frontotemporal dementia; SD = semantic dementia; AD = Alzheimer's disease.

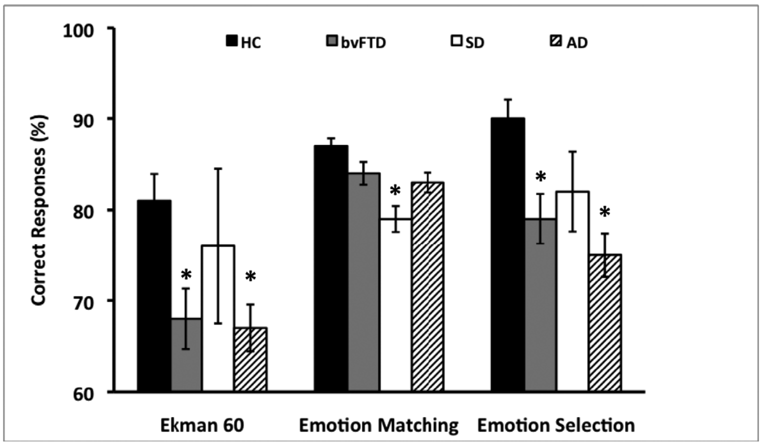

Fig. 2. Results of ANCOVA analyses, which partial out the effects of object naming, face matching and word-picture matching performance respectively. Asterisks indicate which groups remain impaired relative to control subjects ($p < 0.001$).

groups of patients were impaired on tasks of emotion processing, some deficits were no longer present after taking into account performance on tasks of perception, naming or semantic knowledge. These findings support the view that perception or language deficits influence performance on tests of face emotion processing, depending on task demands. Importantly, however, each patient group remained impaired on a subset of the face emotion processing measures, which indicates that patients with AD, bvFTD and SD do exhibit a primary emotion processing disturbance that is not explained by other contributing cognitive processes.

In the SD group, the impairment on the two emotion processing tasks involving verbal labelling of emotional expressions (Ekman 60 and Emotion Selection) was eliminated after covarying performance from the control tasks containing a verbal/semantic component (i.e., BNT 15 and Word-Picture Matching). Hence, SD patients may well be failing emotion processing tasks requiring the production or comprehension of a verbal label as a result of fundamental impairments in language skills. Critically, however, these patients continued to experience a significant impairment on an emotion judgement task with no overt verbal requirements (Emotion Matching), even when a perceptual control task involving identity matching served as a covariate. Like others [18,19], we found that SD patients showed no fundamental perceptual impairment in matching facial identities when emotional expression had to be ignored. Together, these findings indicate that patients with SD have difficulty discriminating different facial emotions, a deficit compounded by their semantic loss. It is likely that this deficit also contributes to their loss of empathy in everyday life (e.g. [40]).

In the present study, only SD patients with predominantly left temporal lobe atrophy were included. This patient selection may account for some of the differences between our results and previous studies. Using a similar approach, Rosen and colleagues [19] reported deficits in face emotion processing in a group of temporal-variant FTD cases even after BNT15 and MMSE scores were covaried. Rosen and colleagues, however, included patients with predominant right temporal lobe atrophy and, in fact, found that right amygdala volume was an important predictor of emotion processing task performance. In other words, the deficits on face emotion labelling tasks may be particularly influenced by language processing deficits in patients with predominantly left temporal lobe atrophy. Right temporal lobe atrophy, on the other hand, seems to cause a deficit on the task that is more related to emotion processing per se.

Patients with bvFTD had difficulties associating emotion labels with facial expressions. This deficit was present both when a particular emotion had to be named and when a face had to be chosen to match a particular verbal label. It is interesting to note that the deficit on this latter task was observed despite the fact that these patients experienced little difficulty on the semantic word-picture matching control task. Previous research has shown that bvFTD patients have difficulties labelling emotional vocalisations [18] and the emotion they would experience in reaction to verbally described scenarios [16]. In contrast, bvFTD patients remain able to define the meaning of an emotion label [16]. In other words, these patients experience a breakdown in their ability to assign appropriate labels to emotional stimuli. This deficit likely impinges on their ability to talk about emotional states and contributes to their characteristic inappropriate behavioural responses.

An unanticipated finding in the bvFTD group was the presence of impaired performance on the face identity matching control task. Given the absence of perceptual difficulties on the Word-Picture Matching task, this finding suggests that these patients have a specific impairment with face perception. Difficulties with face perception (e.g., same/different sex decisions) in bvFTD patients have been reported previously [24], although not consistently [16–18,25]. Face processing is thought to be primarily carried out by posterior brain regions [41], whereas the site of brain atrophy in bvFTD primarily involves anterior brain regions [42], which have been shown to compromise top-down visual spatial processes [43]. In addition, cortical thinning in the inferior parietal region and posterior cingulate in patients with FTD compared to control subjects has recently been reported [30]. It is therefore plausible that the severity of cortical thinning in these brain regions contributes to face perception deficits. We are currently investigating this issue.

Previous research has implicated specific brain regions in the processing of particular emotions (e.g. [1, 2,6–10]). Although the present experiment was not designed to investigate this issue, we have recently completed a different study of emotion recognition and found no clear relationship between FTD subtype and the ability to process specific emotions [44], similar to the results of others [17,18].

Overall, the ability to understand facial emotional expressions is an important component for day-to-day social interactions and several researchers have speculated that this difficulty is at the heart of some of the behavioural abnormalities seen in FTD (e.g. [12,14,24]). Kipps et al. (2009) found that performance on a face emotion recognition measure did not correlate with an activities-of-daily-living score in patients with bvFTD or AD and concluded that the social dysfunction in FTD is multifactorial. In the present study, patients with AD were found to be impaired on all three of the emotion processing tasks and yet, these patients characteristically show fewer changes in behaviour [45]. It seems possible that patients with AD are able to use other emotional cues provided in everyday life (e.g., prosody, body posture, gestures and verbal explanations) or other cognitive skills to guide their responses, whereas patients with FTD appear unable to do so. Socially inappropriate behaviour is a core clinical feature of bvFTD and it seems likely that these patients have additional deficits with impulse control and self-monitoring [46] that contribute to this behaviour.

Our study provides new insight into the cognitive abilities contributing to face emotion processing. In addition to expanding our knowledge of the mechanisms underlying face emotion processing and their breakdown in dementia syndromes, the findings reported here have clinical relevance: understanding the mechanisms underlying emotion processing deficits in dementia syndromes will help design intervention efforts that are tailored to each dementia type, although further research remains necessary in this area.

## Acknowledgements

This project was supported by a National Health and Medical Research Council (NHMRC) of Australia Project Grant (#510106) and by the Australian Research Council (ARC) Centre of Excellence in Cognition and its Disorders. JRH is supported by an ARC Federation Fellowship (#FF0776229). OP is supported by an NHMRC Clinical Career Development Award fellowship (#510184). A portion of this work was presented at the 2010 meeting of the World Federation of Neurology – Research Group on Aphasia and Cognitive Disorders in Istanbul, Turkey.
